# Perspectives of Patients With Orthopedic Trauma on Fully Automated Digital Physical Activity Measurement at Home: Cross-sectional Survey Study

**DOI:** 10.2196/35312

**Published:** 2023-02-09

**Authors:** Julian Scherer, Vithush Yogarasa, Thomas Rauer, Hans-Christoph Pape, Sandro-Michael Heining

**Affiliations:** 1 Department of Traumatology University Hospital of Zurich Zurich Switzerland; 2 Orthopaedic Research Unit University of Cape Town Cape Town South Africa; 3 Faculty of Medicine University of Zurich Zürich Switzerland

**Keywords:** digital, survey, telehealth, follow-up, orthopedic trauma, trauma, attitude, physical activity, rehabilitation, surveillance, surgical procedure, patients, orthopedic

## Abstract

**Background:**

The automated digital surveillance of physical activity at home after surgical procedures could facilitate the monitoring of postoperative follow-up, reduce costs, and enhance patients’ satisfaction. Data on the willingness of patients with orthopedic trauma to undergo automated home surveillance postoperatively are lacking.

**Objective:**

The aims of this study were to assess whether patients with orthopedic trauma would be generally willing to use the proposed automated digital home surveillance system and determine what advantages and disadvantages the system could bring with it.

**Methods:**

Between June 2021 and October 2021, a survey among outpatients with orthopedic trauma who were treated at a European level 1 trauma center was conducted. The only inclusion criterion was an age of at least 16 years. The paper questionnaire first described the possibility of fully automated movement and motion detection (via cameras or sensors) at home without any action required from the patient. The questionnaire then asked for the participants’ demographics and presented 6 specific questions on the study topic.

**Results:**

In total, we included 201 patients whose mean age was 46.9 (SD 18.6) years. Most of the assessed patients (124/201, 61.7%) were male. Almost half of the patients (83/201, 41.3%) were aged between 30 and 55 years. The most stated occupation was a nine-to-five job (62/199, 30.8%). The majority of the participants (120/201, 59.7%) could imagine using the proposed measurement system, with no significant differences among the genders. An insignificant higher number of younger patients stated that they would use the automated surveillance system. No significant difference was seen among different occupations (*P*=.41). Significantly more young patients were using smartphones (*P*=.004) or electronic devices with a camera (*P*=.008). Less than half of the surveyed patients (95/201, 47.3%) stated that they were using tracking apps. The most stated advantages were fewer physician visits (110/201, 54.7%) and less effort (102/201, 50.7%), whereas the most prevalent disadvantage was the missing physician-patient contact (144/201, 71.6%). Significantly more patients with a part-time job or a nine-to-five job stated that data analysis contributes to medical progress (*P*=.047).

**Conclusions:**

Most of the assessed participants (120/201, 59.7%) stated that they would use the automated digital measurement system to observe their postoperative follow-up and recovery. The proposed system could be used to reduce costs and ease hospital capacity issues. In order to successfully implement such systems, patients’ concerns must be addressed, and further studies on the feasibility of these systems are needed.

## Introduction

The surveillance of daily activity and range of motion (ROM) after surgical procedures is still a crucial part of postoperative care and rehabilitation [1]. Furthermore, daily activity serves as a key factor in quality of life assessments, especially in patients with reduced mobility, such as older patients [2]. With the increasing technical developments in recent years, it is now possible to measure not only daily activity via motion sensors but also, for example, the ROM of distinct extremities at home without any active interaction with the patient [3-5]. Several studies have shown the feasibility of health monitoring in private spaces for patients with disabilities who require assistance [6,7]. Recent studies have shown that especially older adults (aged ≥70 years) would be willing to use wearable sensors or have sensors placed at home for monitoring their activities of daily life [8]. This so-called *smart home monitoring* not only can facilitate rehabilitation at home but also may serve as a diagnostic method in the future [9,10]. As a consequence, smart home medical surveillance can be advantageous for both the patient and the whole treatment team; treatment costs can be lowered, and the frequency of visits to a physician’s office, as well as the number of physiotherapists required, can be reduced. Furthermore, this special form of telemedicine can help patients who experience difficulties with accessing the health care system due to the need to travel long distances or a disability [11,12]. Additionally, digital automated home surveillance may be advantageous for postsurgical follow-ups in times when face-to-face appointments are undesirable (eg, during the current COVID-19 pandemic) [13,14]. Postoperative wireless home monitoring after elective joint replacement has been shown to have good satisfaction rates among patients [15]. Since our proposed advanced home monitoring system is not a standard of care, data on the willingness of patients with orthopedic trauma to use these digital systems are lacking. Thus, the aims of this study were to assess the attitudes of patients with orthopedic trauma toward automated digital physical activity surveillance at home and evaluate the participants’ perceptions on the advantages and disadvantages of home surveillance measurements.

## Methods

### Patients and Survey Design

A paper questionnaire was created especially for this cross-sectional survey study and was handed out to outpatients who were treated at the Department of Traumatology of the authors’ institution by nursing staff. The only inclusion criteria were patients who were being treated in the outpatient clinic of the Department of Traumatology and patients who were aged at least 16 years.

The questionnaire (Multimedia Appendix 1) described the possibility of fully automated movement and motion detection (via cameras or sensors) at home without any action required from the patient. For example, daily activity and the ROM of certain extremities could be measured automatically after an operation, and anonymized data (eg, no pictures or videos; ie, data points only) would be sent to the treating physician for further analysis. Specific questions regarding general willingness to undergo automated data acquisition, whether patients were in possession of a smartphone or a laptop, app usage, and the possible advantages and disadvantages of the proposed automated motion tracking system were asked.

In addition, data on the participants’ baseline characteristics, including age, gender, the number of members in their household, and profession, were obtained. At the end of the questionnaire, participants were able to leave a comment on the survey.

### Ethical Considerations

By answering the questionnaire, participants gave consent to the use of the data that they had provided. The institutional ethics committee (Clinical Trial Center of the University Hospital of Zurich) ruled that no formal ethics approval was required.

### Statistical Analysis

Further statistical analyses were performed with the use of SPSS Statistics Desktop 26.0 for Mac (IBM Corp). The data were presented as frequencies (n) and means with SDs. To assess differences in means between 2 groups, an independent samples *t* test was used for normally distributed continuous data, and a chi-square test was used for categorical data. A subgroup analysis was performed for the age (<30 years, 30 to 55 years, and >55 years [arbitrary thresholds]), gender, and occupation subgroups. The level of statistical significance was set at *P*<.05.

## Results

### Demographics

In total, 201 patients (75 female patients and 2 patients of undefined gender) whose mean age was 46.9 (SD 18.6) years were included. Most of the participants (83/201, 41.3%) were aged 30 to 55 years. Most of the patients stated that they were working from 9 AM to 5 PM (ie, an office job; 62/199, 30.8%), 18.9% (38/199) were retired, and 16.9% (34/199) reported “other.” Shift work (25/199, 12.4%), part-time work (25/199, 12.4%), and self-employment (15/199, 7.5%) were the least stated answers. Most of the patients (76/201, 37.8%) were living with 1 other person, and 24.9% (50/201) stated that they were living alone (mean 2.48, SD 1.35; range 0-5).

### Responses to Specific Questions

#### Question 1: Could You Imagine Using a Fully Automated Digital Physical Activity Measurement System at Home?

**Figure 1:**
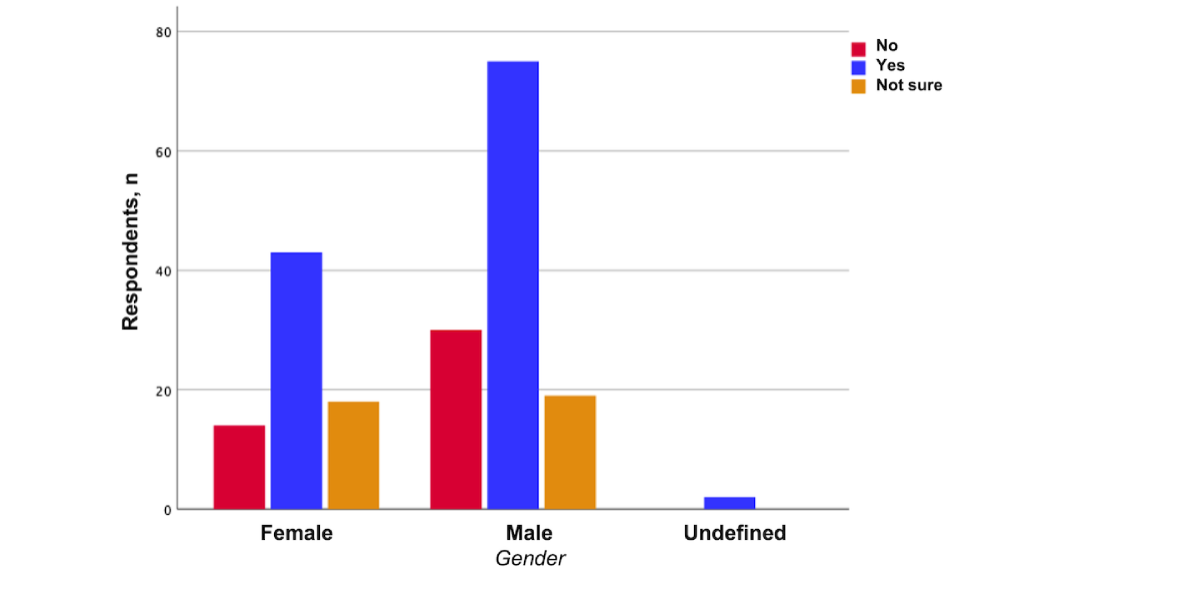
Willingness to use a fully automated digital physical activity measurement system at home (stratified by gender).

**Figure 2:**
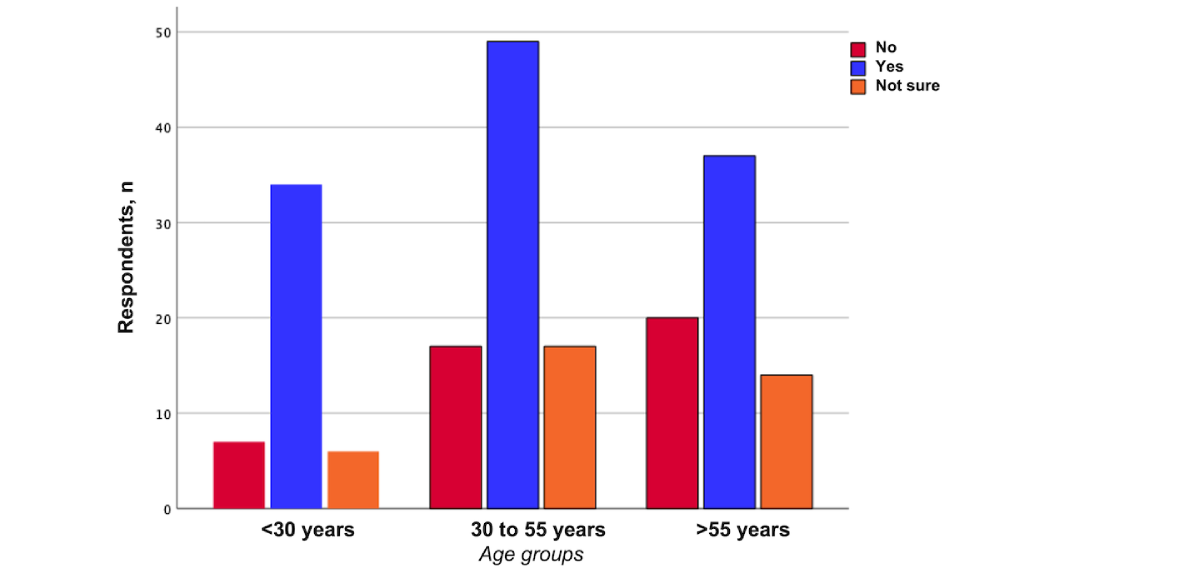
Willingness to use a fully automated digital physical activity measurement system at home (stratified by age groups).

Most patients (120/201, 59.7%) stated that they could imagine using the proposed measurement system, and 18.4% (37/201) were unsure. There was no significant difference between male and female participants (*P*=.41; Figure 1). Patients aged younger than 30 years were insignificantly more likely to use the proposed home surveillance system (34/47, 72%) than patients aged between 30 and 55 years (49/83, 59%) and participants aged older than 55 years (37/71, 52%; *P*=.25; Figure 2). In terms of occupation, there was no statistical difference between the groups regarding their acceptance of the proposed home surveillance system (*P*=.43). The highest acceptance was seen in patients with part-time jobs (17/25, 68%) and patients with nine-to-five jobs (42/62, 68%). The least acceptance was seen in the group of self-employed patients (5/15, 33%; *P*=.43). With regard to the number of persons in participants’ respective households, the highest acceptance was seen in patients living with 4 other people (11/14, 79%), followed by patients living with 1 person (49/76, 65%). The least acceptance was seen in patients living with 2 other people (15/30, 50%; *P*=.56).

#### Question 2: Do You Own a Smartphone?

Most of the participants (188/201, 93.5%) stated that they owned a smartphone. Significantly more young patients (47/47, 100%) owned a smartphone compared to their older counterparts (patients aged 30 to 55 years: 80/83, 96%; patients aged >55 years: 61/71, 86%; *P*=.004).

#### Question 3: Do You Own Any Other Electronic Device With a Camera (eg, Laptop or Tablet)?

Most of the patients (174/200, 86.6%) stated that they owned other electronic devices with a camera. Significantly more young patients (46/47, 98%) owned another electronic device with a camera compared to their older counterparts (patients aged 30 to 55 years: 74/82, 90%; patients aged >55 years: 54/71, 76%; *P*=.008).

#### Question 4: Do You Use Tracking Apps on Your Smartphone (eg, Step Count)?

Most surveyed patients (106/201, 52.7%) stated that they were not using any tracking apps. Young patients used tracking apps insignificantly more often than their older counterparts (*P*=.34). No differences were seen among genders (*P*=.61).

#### Question 5: What Advantages Do You See in the Proposed Automatic Digital Home Surveillance System?

**Figure 3:**
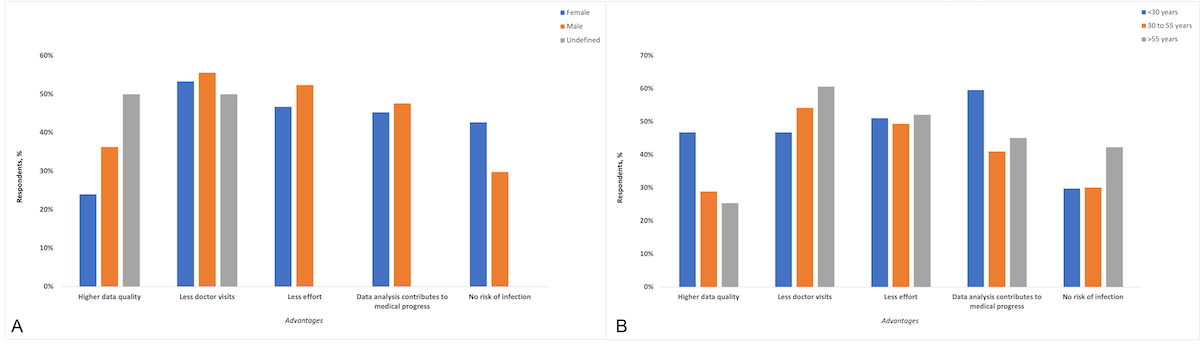
A: Advantages of using the proposed fully automated digital physical activity measurement system at home (stratified by gender). B: Advantages of using the proposed fully automated digital physical activity measurement system at home (stratified by age groups).

The most stated reason for being in favor of the proposed digital home surveillance system was fewer physician visits (110/201, 54.7%), followed by less effort (102/201, 50.7%) and the belief that data analysis contributes to medical progress (94/201, 46.8%). There were no significant differences between male and female participants (*P*=.11). Both male participants and female participants mostly agreed that fewer physician visits were an advantage of the proposed surveillance method (female: 40/75, 53%; male: 69/124, 55.6%; *P*=.94; Figure 3A), but it must be noted that with only 2 patients in the undefined gender group, the assessed answers were insignificant. With regard to the age groups, significantly more young patients stated that higher data quality was an advantage of the proposed system (patients aged <30 years: 22/47, 47%; patients aged 30 to 55 years: 24/83, 29%; patients aged >55 years: 18/71, 25%; *P*=.04; Figure 3B).

Significantly more patients with a part-time job or a nine-to-five job than other surveyed patients stated that data analysis contributes to medical progress and believed that this was an advantage (*P*=.047).

#### Question 6: What Disadvantages Do You See in the Proposed Automatic Digital Home Surveillance System?

The most stated disadvantage of the proposed digital surveillance system was the missing patient-physician contact (144/201, 71.6%), followed by the lack of incidental findings (87/201, 43.3%) and possible measurement errors (77/201, 38.3%). There were no significant differences between male and female participants (*P*=.07).

**Figure 4:**
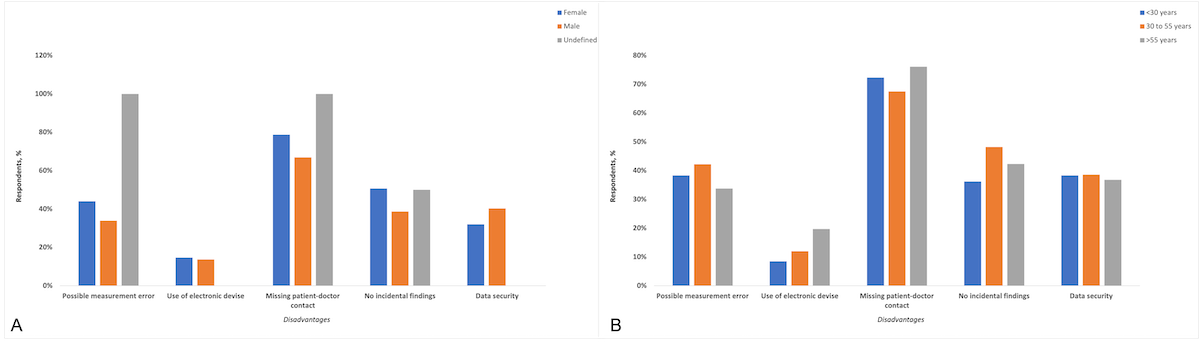
A: Disadvantages of using the proposed fully automated digital physical activity measurement system at home (stratified by gender). B: Disadvantages of using the proposed fully automated digital physical activity measurement system at home (stratified by age groups).

Both male participants and female participants mostly agreed that the missing patient-physician contact was a disadvantage of the proposed surveillance method (female: 59/75, 79%; male: 83/124, 66.9%; *P*=.14; Figure 4A), but it must be noted that with only 2 patients in the undefined gender group, the assessed answers were insignificant. No significant differences were found between the age groups. Within all age groups, the missing patient-physician contact was the strongest disadvantage of using the proposed digital automatic surveillance system at home (patients aged <30 years: 34/47, 72%; patients aged 30 to 55 years: 56/83, 68%; patients aged older than 55 years: 54/71, 76%; *P*=.50; Figure 4B). No significant differences were found between the job groups, but among these groups, the most stated disadvantage was the missing patient-physician contact.

There were no specific answers in the comments section of the questionnaire.

## Discussion

### Principal Findings

The aim of this study was to assess the willingness of patients with orthopedic trauma to conduct so-called *smart home monitoring* after a surgical procedure to observe their postoperative course and physical activity. We also aimed to identify the digital equipment that such patients owned, assess their use of digital equipment, and determine the advantages and disadvantages of the proposed monitoring system. It should be mentioned that the survey was carried out during the current COVID-19 pandemic.

Our study results show that the majority (120/201, 59.7%) of the assessed patients could imagine using the proposed measurement system to observe their postoperative course. Previous studies have shown, for example, that smartphone usage in the context of mobile health (mHealth) has significantly increased in the female population [16]. In our study, there was no significant difference between male and female patients (*P*=.41). The willingness to use an automated digital measurement system at home decreased with age, which could have been due to the lower usage of electronic devices within the older adult population. Several studies have found a decline in digital usage among older patients resulting from their decreased affinity toward digital usage (ie, when compared to their younger counterparts) [17,18]. A recent study has also shown that the usage of mHealth technologies declines with age [19]. In our study, patients aged younger than 30 years had higher acceptance (34/47, 72%) than patients aged between 30 and 55 years (49/83, 59%), although there was no significant difference between these groups (*P*=.25). The participants aged older than 55 years had the lowest acceptance (37/71, 52%), which is contrary to the findings of a previous study where it was shown that especially people aged above 70 years were eager to be monitored for their activities of daily life [8]. There was also no significant difference in acceptance among the different occupations in our study. Part-time jobs and nine-to-five jobs showed the highest acceptance rates, although insignificant (*P*=.43). This could have been due to participants with such jobs being familiar with technology (eg, digital video meetings, calls, etc), especially under the current COVID-19 situation, during which digital instruments have been strongly promoted [20]. We found insignificant differences in acceptance based on the number of persons in participants’ respective households. Participants who were living with 4 other people were more likely to use the measurement system than participants who were living with only 2 other people (*P*=.56). To our knowledge, there is no existing data on the impact of the number of persons living in a household on the acceptance of automated medical surveillance. It seems logical to the authors that postsurgical care could be more difficult for families with more people than, for example, couple households and that such families would prefer digital surveillance by physicians.

We found that most of the participants (188/201, 93.5%) owned a smartphone, with younger patients being more likely to use a smartphone than older patients, which is consistent with data from the United States and the European Union [21,22]. Of the 201 patients, 106 (52.7%) stated that they were not using any tracking apps. To our knowledge, there are no general data on the usage of tracking apps, and we were unable to provide such data, since the individual circumstances of each participant were not assessed.

Most of the surveyed patients stated that fewer physician visits (110/201, 54.7%) and less effort (102/201, 50.7%) were advantages of the proposed surveillance measurement system. Several studies have shown that automated surveillance (eg, via smartphones) can reduce health costs, reduce travel times, and allow health care data to be obtained sufficiently [12,23]. Previous studies have also shown that having to perform fewer actions in surgical aftercare (eg, traveling) is of major importance to patients [13,14]. Significantly more younger patients stated that better data quality would be an advantage of the proposed surveillance system (*P*=.04), which can be seen as younger patients having a better understanding of digital usage and its advantages than older individuals. It can be assumed that in the current COVID-19 pandemic situation, no risk of infection in physicians’ offices is an advantage that has gained importance, as already described in previous studies [13,24]. Less than half of the surveyed patients (69/201, 34.3%) stated that this would be an advantage of the proposed surveillance system, which is a lower rate than those reported in previous studies. However, a higher number of female participants believed that this was an advantage, and this is consistent with the findings of previous studies [13,25,26]. The most frequently stated disadvantages were the missing patient-physician contact and the lack of incidental findings. Not having patient-physician contact was a major concern in the older adult group, as already seen in previous studies [14]. Surprisingly, data security was not a concern for most of the surveyed patients (74/201, 36.8%), which is inconsistent with the findings of previous studies [14,27,28]. To our knowledge, there are no previous studies on the willingness to conduct automated digital surveillance at home postsurgically. Based on the wide acceptance of the proposed automated postsurgical surveillance system seen in our study, this system can be advantageous in the daily clinical routines of both patients and health care workers. This already well-studied alternative can save time and have lower costs. In addition, automatically collected data could be used for quality assessment and research. Having as much data about patients as possible is important for the measurement of daily activity, as such data could improve the quality of postoperative follow-ups.

This study has some limitations. A questionnaire is always directly linked to a participant and how they understand the questions. This could lead to different perceptions of the questionnaire, which could potentially result in bias. In addition, the participants were all patients who were being treated at our hospital, and we did not assess the individual reasons for consultations, which could have biased their opinions on the willingness to be home-monitored postoperatively (eg, injury requiring immobilization). Furthermore, surveys are generally of relatively low study quality. Finally, due to voluntary participation, patients with a negative attitude toward digital solutions might be underrepresented.

### Conclusion

Our results show that most of the assessed participants (120/201, 59.7%) would use the proposed automated digital measurement system to observe their postoperative follow-up and recovery. This system could be used broadly in order to minimize costs and travel times. Furthermore, in times of decreased hospital capacity, an automated home surveillance system could ease the pressure on hospitals by increasing the availability of hospital beds. Data security was not a major concern for most of the participating patients (74/201, 36.8%), which could be a benefit for the implementation of these systems with regard to legal issues. Patients’ concerns must be addressed in order to successfully implement a home surveillance system. Further studies should investigate the practical feasibility of automated home surveillance systems for orthopedic postsurgical follow-up and determine which group of patients would benefit the most from such systems.

## Appendix

Multimedia Appendix 1 Paper questionnaire.

## Abbreviations

mHealth: mobile health
ROM: range of motion

## Data Availability

The data sets generated and analyzed during this study are available from the corresponding author on reasonable request.
