# Supplementary material for: Perspectives of Patients With Orthopedic Trauma on Fully Automated Digital Physical Activity Measurement at Home: Cross-sectional Survey Study
Source: JMIR Form Res. 2023 Feb 9;7:e35312. doi: 10.2196/35312 (PMC9951073; doi:10.2196/35312)
Supplement: Multimedia Appendix 1 [file formative_v7i1e35312_app1.pdf]

The automatized digital measurement of daily activity at home

The digital knowledge is developing, and it’s being used more and more in medicine. Usually, a surgical treatment consists of several post-operative follow-ups, these have to be done in the hospital in the presence of the doctor. Soon, data like the mobility of the patient after a surgical treatment could be collected directly from their home through an automatized digital device (the patient doesn’t need to do anything for it). Sensors or a video analysis could be used for this purpose. The collected data will be coded and sent anonymously to the treating doctor (no visual material will be saved).

With this survey we want to know your opinion on this automatized digital measurement as a patient.

Thank you for taking part in this survey. The participation is voluntary and contains the enclosed survey below (ca. 5 mins). Your data will be exported in a statistical software, it will not be passed on to third parties and it will be used only for the purpose of this study. By filling in the questionnaire you will give your consent.

If you have any questions don’t hesitate to contact us by e-mails.

With kind regards

General Questions:

1. Age: ______ years

2. Sex: ☐ woman ☐ man ☐ diverse

3. Working hours: ☐ shift work ☐ part time ☐ self-employed
☐ office time (09:00-17:00) ☐ pensioner
☐ other

4. Persons in household: ☐ 1 Pers. ☐ 2 Pers. ☐ 3 Pers.
☐ 4 Pers. ☐ 5 Pers. ☐ > 5 Pers.

Specific questions:

1. Would you use such an automatized digital device at home?
☐ yes ☐ no ☐ unsure

2. Do you use a smartphone?
☐ yes ☐ no ☐ unsure

3. Do you use another electrical device with a camera (e.g., laptop, tablet)?
☐ yes ☐ no ☐ unsure

4. Do you use a “tracking app” (e.g., step counter on your smartphone)?
☐ yes ☐ no ☐ unsure

5. Where do you see advantages in an automatized digital device?
☐ higher data quality
☐ fewer hospital visits
☐ less effort for the patient
☐ the data analysis contributes to the medical progress
☐ no general risk for any infections (e.g. approach with public transport, hospitalization)
☐ other: ____
____
____

6. Where do you see disadvantages in an automatized digital device?
☐ measuring mistake
☐ usage of electronical device
☐ lack of doctor-patient contacts
☐ no incidental findings possible (e.g., during a physical examination)
☐ problems with data protection
☐ other: ____
____
____

7. Other comments:
____
____
____
____
____

Thank you for your participation!
